# Brain CT registration using hybrid supervised convolutional neural network

**DOI:** 10.1186/s12938-021-00971-8

**Published:** 2021-12-29

**Authors:** Hongmei Yuan, Minglei Yang, Shan Qian, Wenxin Wang, Xiaotian Jia, Feng Huang

**Affiliations:** 1 Neusoft Research of Intelligent Healthcare Technology, Co. Ltd, A1 Building, No.2 Xinxiu Street, Hunnan New District, Shenyang, 110179 People’s Republic of China; 2 Neusoft Medical System, Co. Ltd, Shenyang, 110167 China; 3 Shenyang Advanced Medical Equipment Technology Incubation Center, Co. Ltd, Shenyang, 110167 China

**Keywords:** Image registration, Brain CT, Intersubject, Deep learning, Hybrid supervision

## Abstract

**Background:**

Image registration is an essential step in the automated interpretation of the brain computed tomography (CT) images of patients with acute cerebrovascular disease (ACVD). However, performing brain CT registration accurately and rapidly remains greatly challenging due to the large intersubject anatomical variations, low resolution of soft tissues, and heavy computation costs. To this end, the HSCN-Net, a hybrid supervised convolutional neural network, was developed for precise and fast brain CT registration.

**Method:**

HSCN-Net generated synthetic deformation fields using a simulator as one supervision for one reference–moving image pair to address the problem of lack of gold standards. Furthermore, the simulator was designed to generate multiscale affine and elastic deformation fields to overcome the registration challenge posed by large intersubject anatomical deformation. Finally, HSCN-Net adopted a hybrid loss function constituted by deformation field and image similarity to improve registration accuracy and generalization capability. In this work, 101 CT images of patients were collected for model construction (57), evaluation (14), and testing (30). HSCN-Net was compared with the classical Demons and VoxelMorph models. Qualitative analysis through the visual evaluation of critical brain tissues and quantitative analysis by determining the endpoint error (EPE) between the predicted sparse deformation vectors and gold-standard sparse deformation vectors, image normalized mutual information (NMI), and the Dice coefficient of the middle cerebral artery (MCA) blood supply area were carried out to assess model performance comprehensively.

**Results:**

HSCN-Net and Demons had a better visual spatial matching performance than VoxelMorph, and HSCN-Net was more competent for smooth and large intersubject deformations than Demons. The mean EPE of HSCN-Net (3.29 mm) was less than that of Demons (3.47 mm) and VoxelMorph (5.12 mm); the mean Dice of HSCN-Net was 0.96, which was higher than that of Demons (0.90) and VoxelMorph (0.87); and the mean NMI of HSCN-Net (0.83) was slightly lower than that of Demons (0.84), but higher than that of VoxelMorph (0.81). Moreover, the mean registration time of HSCN-Net (17.86 s) was shorter than that of VoxelMorph (18.53 s) and Demons (147.21 s).

**Conclusion:**

The proposed HSCN-Net could achieve accurate and rapid intersubject brain CT registration.

## Background

Over the past several decades, the incidence and mortality of acute cerebrovascular disease (ACVD) in China has steadily increased, among which acute ischemic stroke (AIS) has become one of the leading cause of death. Proper treatment at the acute stage is of great importance for favorable outcomes [1]. Computed tomography (CT) scans have become one of the significant and routine examinations for ACVD because of its convenience and quickness [2]. CT images also have considerable advantages in terms of hospital deployment and clinical utilization rate [3]. It is routinely adopted to evaluate early brain ischemic changes in the clinic, where the Alberta Stroke Program Early CT Score (ASPECTS) is commonly recommended. The middle cerebral artery (MCA) blood supply area is the main quantitative area of ASPECTS.

The performance of automatic ASPECTS depends heavily on brain CT registration between the patient and the atlas. However, automatic and rapid intersubject brain CT registration remains a great challenge mainly due to the following reasons: first, the human brain morphology is highly complex; second, the soft-tissue resolution of the brain CT images is relatively low; third, the structural contrast of gray/white matter in brain CT images is relatively low; fourth, large anatomical structural variations may exist across individuals; finally, three-dimensional (3D) brain CT volume registration has a heavy computational cost.

Most traditional brain registration algorithms could be summed up as iterative optimization problems. High-dimensional optimization might lead to a heavy computational burden and slow convergence speed. The common registration time for 3D brain CT images was generally as high as tens of minutes [4–10]. For example, when using B-spline as the elastic deformation model of a registration method, the image should be gridded first, and then, B-spline could be used to control grid changes to fit the whole image transformation [11]. These methods could accurately simulate local deformations [12], but the running speeds were very slowly and thus could not easily meet the clinical interpretation needs for acute brain CT images. Demons-based registration methods are another type of nonrigid algorithms that can be used for brain image registration. They use an optical flow field to model image elastic deformation and iteratively optimize the deformation field. These algorithms are based on gray information and thus can avoid manual interference in feature extraction. However, they are suitable only for small deformations and are sensitive to image gray variations [13].

Recently, deep learning-based registration methods have been proposed for accurate and fast medical image registration [14–16]. VoxelMorph [17], a deep learning framework for brain magnetic resonance (MR) proposed by Dalca et al., applies convolutional neural networks (CNNs) for unsupervised image registration. A full CNN method for brain MRI registration [18] proposed by Fan et al. utilizes the double supervised training method. Han et al. proposed a deformable MR–CT brain registration method [19] that first synthesizes a CT image from MR and then registers the synthetic CT to the intraoperative CT with an inverse-consistent registration network. Current brain registration networks are often used for monomodal MR image registration and multimodal image registration (MR and CT), whereas intersubject brain CT–CT image registration has rarely been reported [20].

Previous studies have shown that deep learning-based registration algorithms have improved registration speeds, but provide slightly worse registration results than traditional algorithms. Furthermore, overcoming the challenge of large intersubject anatomical deformation remains difficult [21, 22]. Moreover, although supervised learning-based neural networks can theoretically improve registration accuracy, the difficulty of obtaining high-quality registration gold standards is the most challenging problem that limits the practical applications of supervised learning-based neural networks [23].

To solve the above problems, a fully automatic registration method based on a hybrid supervised CNN (HSCN-Net) was proposed for accurate and fast intersubject brain CT registration to improve the interpretation of images from patients with ACVD. The main contributions of this study are summarized as follows:

1. The lack of gold standards is the major challenge encountered in medical image registration with supervised deep learning. Currently, there exist two ways to obtain the gold standards: annotation by neuroradiologists and generation by traditional registration algorithms. However, accurately annotating the displacements of corresponding pixel points in images is difficult. Meanwhile, the accuracy of the gold standards is limited by the traditional registration method used. HSCN-Net applies a simulator to generate synthetic deformation fields and images during training as gold standards, which could improve the model’s registration accuracy, to solve the above problems. We applied the multiple deformation fields generated by the simulator on all the images in the database and asked senior neuroradiologists to check whether the organizational structures in the deformed images conform to the actual changes in brain tissues to verify the reasonableness of the simulations.
2. The existing networks easily fall into the local minimum values and are prone to acquiring inaccurate deformations. In particular, the prediction accuracy is obviously reduced when the intersubject anatomical deformation is large [24]. Large deformations with appropriate proportions are added to the deformation fields generated by the simulator to mitigate this problem. In addition, the simulated deformation fields include a variety of transformations, such as affine and elasticity. Through continuous iterative learning, HSCN-Net provides prediction results that are consistent with the transformation characteristics of real-image pairs, and its capability to handle large intersubject deformation is considerably enhanced.
3. HSCN-Net adopts a hybrid supervised loss function that utilizes the alignment accuracy of the deformation fields as the supervised target and image similarity as the self-supervised target to improve the registration accuracy and generalization capability of the model. The correlation measure of the deformation fields directly quantifies the alignment accuracy of the simulator-generated deformation fields and the predicted deformation fields. This approach could improve the accuracy of the predicted deformation fields. Image similarity is powerful feedback for the registration visualization results and could well ensure the generalization capability of the model.
4. Qualitative and quantitative analyses were performed to assess the performance of the proposed method comprehensively. HSCN-Net could achieve accurate and fast brain CT image registration, and addresses the scarcity of excellent algorithms for brain CT image registration. It was helpful for improving and accelerating the interpretation of CT images from patients with ACVD, thereby assisting clinicians in acute diagnosis and treatment decision-making.

## Results

### Qualitative analysis

**Fig. 1:**
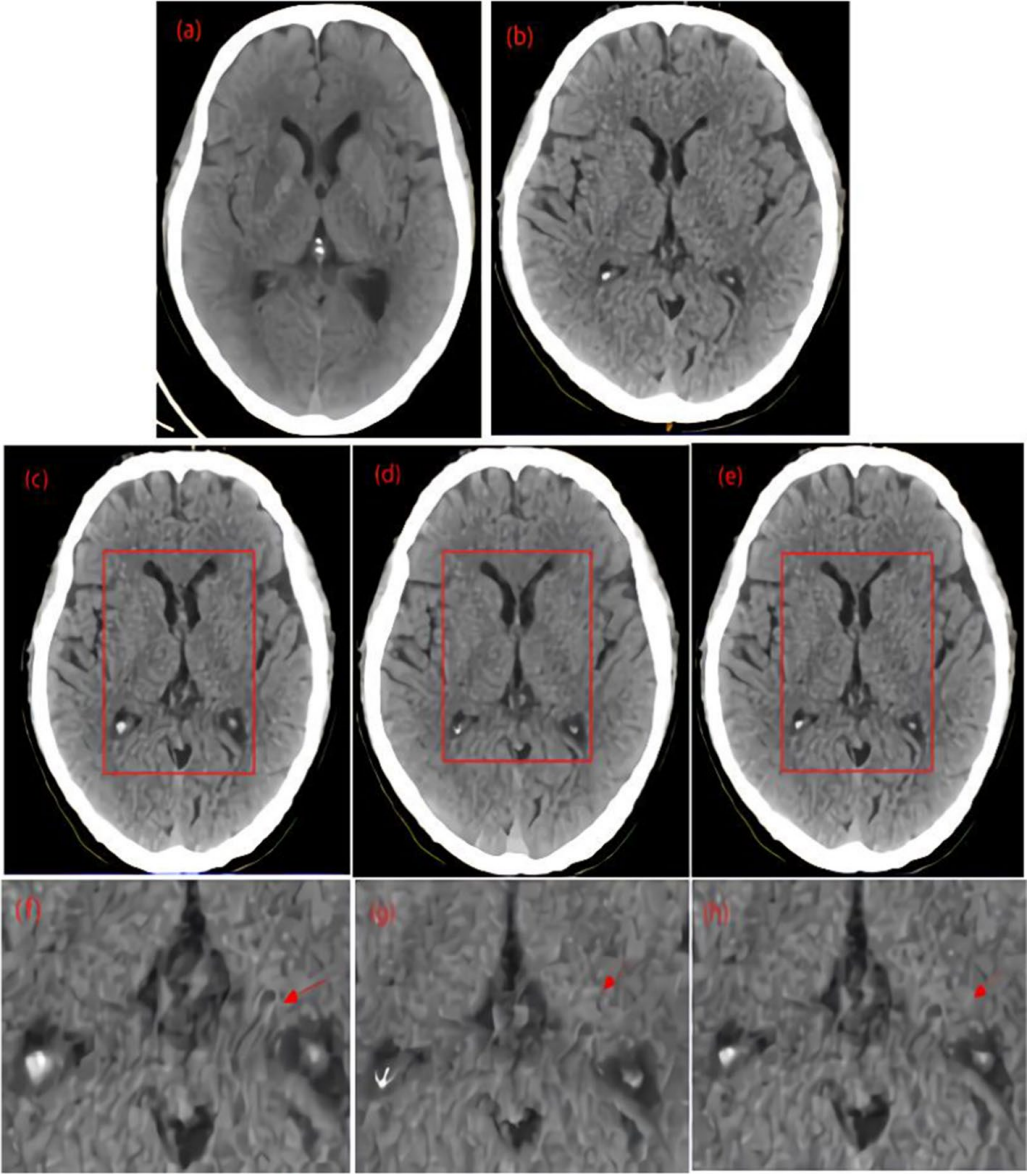
Registration results of a typical brain CT image: **a** is the reference image; **b** is the moving image; and **c**–**e** are the registration results of HSCN-Net, Demons, and VoxelMorph algorithms, respectively; **f**–**h** are the local enlarged images corresponding to the same parts in **c**–**e**. All the three registration methods can achieve spatial matching. Compared with the ventricle in **e**, that in **c** and **d** had better spatial matching. The deformations in **f** and **h** were smoother than those in **g** and were highly consistent with the smooth characteristics of tissue deformation

It depicted the cross-section visualization of the registration results for a typical case in Fig. 1a is the reference image; (b) is the moving image; and (c)–(e) are the registration results of HSCN-Net, Demons, and VoxelMorph algorithms, respectively. The main part of the rectangular box is the ventricular structure. The ventricular structures in images (c), (d), and (e) were more consistent spatially with those in the reference image (a) than those in the moving image (b). This result indicated that all the three registration methods could achieve spatial matching. Moreover, the size and shape of the ventricles in (c) and (d) were more similar and closer to the corresponding region in the reference image (a) than to those of the ventricle in (e). This result showed that (c) and (d) had good spatial matching. In addition, the deformations between the voxels in image (e) were relatively small. (f)–(h) are the local enlarged images corresponding to the same parts in (c)–(e). As indicated by the arrow, the deformations in (f) and (h) were smoother than those in (g) and were highly consistent with the smooth characteristics of tissue deformation.

### Quantitative analysis

The results of endpoint error (EPE) between the predicted sparse deformation vectors and gold-standard sparse deformation vectors, image normalized mutual information (NMI), Dice, and the running times of the three algorithms are provided in Table 1.

**Table 1:** Registration error and performance quantification table (mean ± standard deviation)

Method	EPE (mm)	NMI	Dice (%)	Time (s)
Demons	3.47 ± 1.03	0.84 ± 0.02	0.90 ± 0.03	147.21 ± 27.46
VoxelMorph	5.12 ± 1.72	0.81 ± 0.02	0.87 ± 0.05	18.53 ± 0.25
HSCN-Net	3.29 ± 0.98	0.83 ± 0.01	0.96 ± 0.04	17.86 ± 0.23

Table 1 shows that the mean EPE values of the three algorithms followed the ascending order of HSCN-Net, Demons, and VoxelMorph. This ranking indicated that HSCN-Net had the smallest deformation field error among the algorithms. Specifically, the EPE mean value of HSCN-Net was 1.83 mm less than that of VoxelMorph. This result strongly indicated that the registration accuracy of HSCN-Net was considerably higher than that of VoxelMorph (*p* < 0.05). The mean NMI of HSCN-Net (0.83) was slightly lower than that of the Demons algorithm (0.84, *p* > 0.05), but was slightly higher than that of the VoxelMorph algorithm (0.81, *p* < 0.05). This result illustrated that the registration results of Demons, followed by those of HSCN-Net, had the highest correlation with the reference image among those of the three registration methods. Furthermore, the Dice coefficient of the MCA blood supply area between the HSCN-Net registration result and the reference image was 0.96, which was higher than that of the Demons (0.90, *p* < 0.05) and VoxelMorph (0.87, *p* < 0.05) algorithms. Only the Dice metric of HSCN-Net was 0.01 higher than that of Demons, and the metric of HSCN-Net had the lowest standard deviation among those of the three methods. This result indicated that HSCN-Net had high robustness and generalization capability. Finally, Table 1 shows that Demons took 147.21 ± 27.46 s, VoxelMorph 18.53 ± 0.25 s, and HSCN-Net 17.86 ± 0.23 s for one case. These results demonstrated that the registration speed of HSCN-Net was slightly higher than that of the classic registration network VoxelMorph (*p* > 0.05) and was considerably higher than that of the traditional registration algorithm Demons (*p* < 0.05).

## Discussion

HSCN-Net, a nonrigid image registration algorithm based on a hybrid supervised CNN for intersubject CT–CT image registration, was proposed in this study. HSCN-Net used a simulator to generate multiscale virtual deformation fields during training to overcome the difficulty in obtaining high-quality deformation fields as the gold standards in supervised learning. Large deformations with appropriate proportions were added to the generated deformation fields to meet the challenge of large intersubject anatomical deformation between the CT images to be registered. Moreover, the proposed HSCN-Net included a hybrid supervised loss function that combines the multiple information of deformation field and image similarity. This function significantly improved the accuracy and generalization capability of the network. A total of 101 CT images were collected for model construction and model assessment. The results of qualitative and quantitative assessments revealed that the proposed method could achieve accurate and rapid CT–CT image registration in the presence of large intersubject anatomical deformations.

Given that the supervised learning-based registration network could provide the deformation fields that corresponded to the registration images, the network could learn the information of the prediction deformation fields directly under supervision. In contrast to the unsupervised learning-based network, the supervised learning-based registration network could simplify network training and improve registration accuracy in theory. However, the supervised learning method required high-quality golden standards to supply supervision information, and accurately annotating the displacements of the corresponding pixel points in medical images with a large number of pixels was burdensome. Completing the tedious and time-consuming annotation is difficult even for senior neuroradiologists. An alternative method was to use a traditional method to generate deformation fields as the gold standards. However, the accuracy of the gold standard was limited by the registration accuracy of the traditional method used. Therefore, in the practice network training stage, obtaining accurate and large amounts of deformation field gold standards was complicated and is the main reason for the nonideal registration results of supervised learning-based networks [15–17]. HSCN-Net provided an effective, accurate, and convenient way to obtain the deformation field gold standards for the supervised learning-based registration network to solve this problem. First, we used the simulator to generate a virtual deformation field. Then, we applied the produced deformation field on the moving image to generate a virtual deformed image. Finally, we obtained the accurate deformation field between the moving image and reference image for model training. In this way, the accuracy of HSCN-Net was theoretically improved. The reduction in the EPE mean of HSCN-Net by 1.83 mm (*p* < 0.05) relative to that of the unsupervised registration network VoxelMorph verified that HSCN-Net had significantly improved registration accuracy.

Given that many possible transformations of the moving images yielded close similarity measurement values, the gradient of the similarity measurement was insufficiently accurate in the representation of the transformation type and scale in iterative network optimization. In addition, optimization without the initialized transformation at the finest resolution was difficult due to the large degrees of freedom of the transformation parameters. Therefore, existing networks easily falled into the local minimum values and were prone to obtaining inaccurate deformation [24]. In particular, when the intersubject deformation was large, the prediction accuracy was obviously reduced [24]. For example, further improving the registration accuracy of VoxelMorph was difficult, and performance worsens in the presence of large intersubject deformation. The large-scale plausible deformations that are produced by HSCN-Net are used directly in the training process to address this challenge. The iterative learning of multiscale deformation made HSCN-Net’s prediction of the deformation field between images highly realistic and suitable for large intersubject deformation. Moreover, the simulated deformation fields included a variety of transformations, such as affine and elasticity transformations. The consistency of the diversity of the transformations with the transformation characteristics of real-image pairs increased, such that the physicality of the synthetic deformation fields increases. This situation further improved the registration accuracy of HSCN-Net. Moreover, the EPE standard deviation of HSCN-Net was 0.74 lower than that of VoxelMorph (*p* < 0.05). This result demonstrated that the generalization capability of HSCN-Net had been effectively improved compared with that of the VoxelMorph algorithm.

Considering that a neural network optimizes parameters by minimizing the loss function, the loss function is one of the key factors affecting the performance of neural networks. In this work, a new loss function that combined the deformation field EPE and image similarity was proposed. The EPE measure directly quantified the alignment accuracy of the deformation fields generated by the simulator and the predicted deformation fields. Such an approach could improve the accuracy of the predicted deformation fields. Image similarity, as a self-supervised training target, reflects the similarity between the reference image and the prediction image. It is powerful feedback for the registration visualization results and can well ensure the generalization capability of the model. Table 1 shows that the NMI mean of the HSCN-Net’s correlation metric had improved by 0.02 compared with that of VoxelMorph (*p* < 0.05), and the overlap degree (Dice coefficient) of the MCA blood supply area between the reference image and the prediction image had improved by 0.09 (*p* < 0.05). Finally, EPE and NMI had the lowest standard deviations, indicating that HSCN-Net had higher robustness and generalization capability than the other algorithms.

Most traditional registration algorithms suffered from slow convergence speed, because they involve high-dimensional iterative optimization [6, 24]. Long running time limited the practical application of many excellent registration algorithms, especially in the diagnosis of some acute diseases, such as ACVD, which required fast CT image interpretation for emergency treatment. The prognosis of the patient was affected seriously if an excessively long time is spent on the inspection or decision-making stage [1]. A previous work [24] proposed a neural network architecture based on Laplacian Pyramid for the evaluation of brain MRI images. The average registration time of the Laplacian Pyramid networks was approximately 0.3 s, which was significantly lower than that of Demons (approximately 190 s). NiftyReg [25] was another commonly used traditional medical image registration method. In the previous work [6], NiftyReg and VoxelMorph-based network were used simultaneously for the registration of longitudinal abdominopelvic CT images. Compared with that of NiftyReg, the registration speed of the VoxelMorph-based network was approximately 300 times faster. Similar conclusions could be found in the present work. Table 1 shows that compared with that of the traditional registration method Demons, the average registration time of HSCN-Net had decreased from 147.21 to 17.86 s. This reduction greatly improved registration speed (*p* < 0.05). Moreover, compared with that of the classic neural network VoxelMorph, the average registration time of HSCN-Net had decreased by 0.67 s (*p* < 0.05). The execution time of the proposed method was considerably shorter than that of Demons and slightly shorter than that of VoxelMorph. These results indicated that HSCN-Net had certain advantages in running speed. Therefore, HSCN-Net has great clinical application value for ACVD, which requires rapid image registration.

Nevertheless, this study had some limitations. First, all the brain CT images involved this work were collected from the one medical center. Currently, CT images could not be collected from multiple manufacturers and equipment. In the future, we will pay additional attention to diverse data sources; continue to collect brain CT data for the optimization and validation of HSCN-Net; and further improve the accuracy, robustness, and generalization capability of HSCN-Net. Second, due to the limited memory resources of the GPU, HSCN-Net cannot be very deep, and random pairs were selected from the training set with a batch size limited to 1. Besides, we will attempt to apply HSCN-Net on CT data from other body parts, such as the heart and liver, for image interpretation.

## Conclusion

A nonrigid algorithm based on a hybrid supervised CNN was proposed for intersubject brain CT image registration. It could achieve accurate and fast brain CT registration with high generalization capability in the presence of large intersubject deformations. This method could help accelerate the evaluation and interpretation of ACVD CT images to provide pieces of evidence to neurologists for acute diagnosis and treatment decision-making.

## Method

### Data set and experimental configuration

Given the absence of published standard datasets for brain CT registration, we used data that were continuously collected from a hospital. Senior neuroradiologists manually annotated the MCA blood supply area in all images of the testing set. A total of 101 nonenhanced CT images were collected as the data set. These images were taken from 45 women and 56 men. The women were aged 30–86 years (71 ± 12 years old), and the men were aged 28–81 years (62 ± 11 years). Of the images, 57 were used for HSCN-Net training, 14 were used for validation, and 30 were used for testing. The data size was 512 × 512 × 64, and the voxel spacing was 0.36 mm × 0.36 mm × 2 mm. All the data were preprocessed via skull removal and Z-score normalization. Our experiment was run on a Win10 64-bit operating system and GeForce RTX 2080 Ti, and the virtual environment was configured in Python 3.6 + TensorFlow 2.0.0.

### Network design

**Fig. 2:**
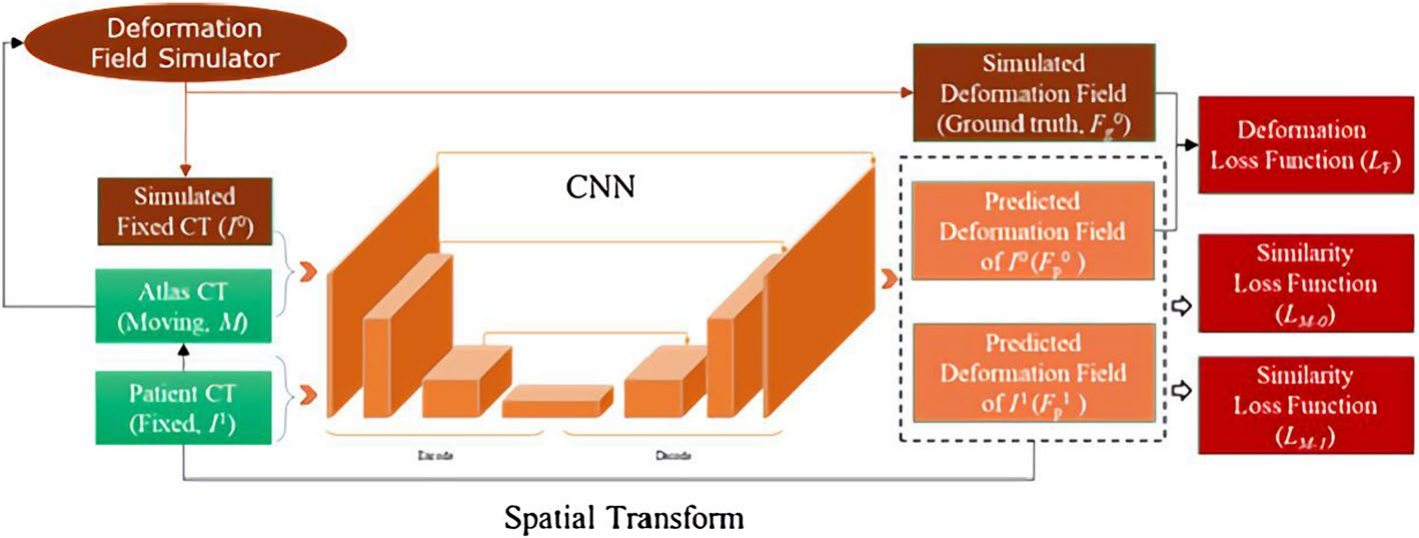
Framework of HSCN-Net. We used a simulator to generate synthetic deformation fields and images during training to address the lack of registration gold standards. Large deformations of appropriate proportions were added to the synthetic deformation fields to overcome the challenge of large deformation fields between the images to be registered. HSCN-Net adopts a hybrid supervised loss function that is constituted by the alignment accuracy of the deformation fields and image similarity to improve the registration accuracy and generalization capability of the model

We propose a Unet-based [26] neural network structure that mainly consists of two parts: the encoding path and decoding path. The encoding path follows the typical architecture of a convolutional network. It is mainly composed of repeated convolution and max pooling operations. The core function of the encoding path is to learn the deep semantic features of images. The decoding part fuses the semantic feature layers obtained in the encoding stage, and every step in the decoding path consists of an up-sampling of the feature map. The network adds dilated convolution inside the encoding and decoding module. This approach can expand the receptive field to improve feature extraction capability without increasing parameter number. The framework of the proposed network is shown in Fig. 2. Importantly, a deformation field simulator is integrated into the network structure. The simulator simulates and generates the gold standards of the deformation fields during training, thus alleviating the difficulty encountered in obtaining high-quality gold standards in supervised learning. The deformation fields are configured intentionally with a certain proportion of large deformations to improve the capability of HSCN-Net to process large deformations. The moving image and the reference image that are deformed on the basis of the moving image by the simulated deformation field are defined as the first set of input images. Next, by leaving the moving image unchanged, another reference image is randomly selected from the training set. These two images are defined as the second set of input images. Then, these two sets of images are input into the network for training at the same time. Finally, deformation field information and image information are obtained simultaneously to optimize the network. Sparse deformation fields are preconstructed on the basis of the anatomical feature structural points in all images of the validation set and used as the gold standard for evaluation in the same way as the testing set discussed in “Method for the evaluation of network performance” section to verify the effect of the network in the training stage.

The network adopts supervised learning and self-supervised learning to optimize network parameters. Training images are divided into the supervised group and the self-supervised group. For the supervised group, the simulator generates the deformation field ${F}_{g}^{0}$ to warp the image $M$ into the reference image ${I}^{0}$. The two images in the self-supervised group, which are denoted as the reference image ${I}^{1}$ and the moving image $M$, are randomly selected from the training set. The two sets of images are simultaneously input into the network for training. Then, the two predicted deformation fields are obtained: ${F}_{p}^{0}$ and ${F}_{p}^{1}$. Three sub-loss functions need to be calculated: the similarity measure ${L}_{F}$ between the random deformation field ${F}_{g}^{0}$ and the predicted deformation field ${F}_{p}^{0}$, the similarity measure ${L}_{M0}$ between the reference image ${I}^{0}$ and the predicted image ${I}_{p}^{0}$ in the supervised group, and the similarity measure ${L}_{M1}$ between the reference image ${I}^{1}$ and the predicted image ${I}_{p}^{1}$ in the self-supervised group. Furthermore, three sub-loss functions are multiplied by the corresponding weight coefficients and added together to acquire the hybrid supervised loss function. Subsequently, the hybrid supervised loss function is fed back into the network backward propagation to optimize the parameters until convergence. Specifically, the hybrid loss function is computed as follows:

1 $${L}_{reg}\left({F}_{p}^{0},{F}_{g}^{0},{I}^{0},{{I}^{1},I}_{P}^{0},{I}_{P}^{1};\theta \right)= {\alpha L}_{F}\left({F}_{p}^{0},{F}_{g}^{0};\theta \right)+{\beta L}_{M0}\left({I}^{0},{I}_{p}^{0};\theta \right)+{\gamma L}_{M1}\left({I}^{1},{I}_{p}^{1};\theta \right),$$

where ${I}_{p}^{0}$ represents the predicted registration image generated by the deformation field ${F}_{p}^{0}$ acting on the moving image $M$, ${ I}_{p}^{1}$ represents the predicted registration image generated by the deformation field ${F}_{p}^{1}$ acting on the moving image $M$, and $\theta$ is the parameter of the neural network. The hybrid supervised loss ${L}_{reg}\left({F}_{p}^{0},{F}_{g}^{0},{I}^{0},{{I}^{1},I}_{P}^{0},{I}_{P}^{1};\theta \right)$ combines multiple information on the accuracy of the deformation field alignment and image similarity, and $\alpha$, $\beta$, $\gamma$ are the hyperparameters for balancing the three sub-losses

2 $${ L}_{F}\left({F}_{p}^{0},{F}_{g}^{0};\theta \right)= \frac{1}{|\Omega |}\sum_{p\in\Omega }{\Vert {F}_{p}^{0}\left(p\right)-{F}_{g}^{0}(p)\Vert }_{{L}_{2}},$$

where $p$ is the voxel position in the image coordinate space $\Omega$. $\left|\Omega \right|$ represents the voxel number in the registration image. The supervised learning-based loss function ${L}_{F}$ quantifies the deviation error between the gold-standard deformation field ${F}_{g}^{0}$ and the predicted deformation field ${F}_{p}^{0}$ to improve the alignment accuracy of the two deformation fields

3 $${ L}_{M0}\left({I}^{0},{I}_{p}^{0};\theta \right)=-\frac{1}{\left|\Omega \right|}\sum_{p\in\Omega }\frac{{\left({\sum }_{{p}_{i}}\left({I}^{0}\left({p}_{i}\right)-\overline{{I}^{0}\left(p\right)}\right)\left({I}_{p}^{0}\left({p}_{i}\right)-\overline{{I}_{p}^{0}\left(p\right)}\right)\right)}^{2}}{{\sum }_{{p}_{i}}{\left({I}^{0}\left({p}_{i}\right)-\overline{{I}^{0}\left(p\right)}\right)}^{2}{\sum }_{{p}_{i}}{\left({I}_{p}^{0}\left({p}_{i}\right)-\overline{{I}_{p}^{0}\left(p\right)}\right)}^{2}},$$

where ${p}_{i}$ is the voxel coordinate in the neighborhood centered on the voxel $f(x,y,z)$. $\overline{{I}^{0}(f(x,y,z))}$ and $\overline{{I}_{p}^{0}(f(x,y,z))}$ are the local means within the window around the voxel position $f\left({x}_{i},{y}_{i},{z}_{i}\right)$ in ${I}^{0}$ and ${I}_{p}^{0}$, respectively

4 $${L}_{M1}\left({I}^{1},{I}_{p}^{1};\theta \right)= -\frac{1}{\left|\Omega \right|}\sum_{p\in\Omega }\frac{{\left({\sum }_{{p}_{i}}\left({I}^{1}\left({p}_{i}\right)-\overline{{I}^{1}\left(p\right)}\right)\left({I}_{p}^{1}\left({p}_{i}\right)-\overline{{I}_{p}^{1}\left(p\right)}\right)\right)}^{2}}{{\sum }_{{p}_{i}}{\left({I}^{1}\left({p}_{i}\right)-\overline{{I}^{1}\left(p\right)}\right)}^{2}{\sum }_{{p}_{i}}{\left({I}_{p}^{1}\left({p}_{i}\right)-\overline{{I}_{p}^{1}\left(p\right)}\right)}^{2}},$$

where ${p}_{i}$ is the voxel coordinate in the neighborhood centered on the voxel $f(x,y,z)$. $\overline{{I}^{1}(f(x,y,z))}$ and $\overline{{I}_{p}^{1}(f(x,y,z))}$ are the local means within the window around the voxel position $f\left({x}_{i},{y}_{i},{z}_{i}\right)$ in ${I}^{1}$ and ${I}_{p}^{1}$, respectively.

The experiment showed that when *α* = 1/13, *β* = *γ* = 0.4, the contributions of the three sub-loss functions ${L}_{F}\left({F}_{p}^{0},{F}_{g}^{0};\theta \right)$, ${L}_{M0}\left({I}^{0},{I}_{p}^{0};\theta \right)$, and ${L}_{M1}\left({I}^{1},{I}_{p}^{1};\theta \right)$ to network parameter optimization were relatively balanced. The similarity loss function ${L}_{F}$ of the supervised learning-based deformation field directly reflects the deviation of the gold-standard deformation field and the predicted deformation field. Such a function is beneficial for improving the accuracy of the network. The self-supervised learning-based image similarity loss functions ${L}_{M0}$ and ${L}_{M1}$ can reduce the dependence of the registration network on the diversity of the training set and help improve the generalization capability of the model.

The simulator randomly generates the deformation field ${F}_{g}^{0},$ including affine and elastic, to model various transformations (indicating anatomical variations across individuals) fully. ${F}_{g}^{0}$ is applied on the moving image $M$ to generate the reference image ${I}^{0}$, such that ${F}_{g}^{0}$ can be the gold standard for warping $M$ into ${I}^{0}$. As the gold standard, ${F}_{g}^{0}$ overcomes the problems of the low accuracy and poor consistency of the training gold standard in supervised learning. Furthermore, the random generation of some large deformations in the deformation field ${F}_{g}^{0}$ alleviates the incapability of the existing registration methods to adapt to large deformations.

Many efficient optimization algorithms [27–30] have been proposed. In this work, we used Adam [29] with default settings as the optimizer. Two measures were adopted to overcome the network overfitting problem. First, the input images of the network were randomly added with Gaussian noise for data augmentation. Second, some dropout units were added to the registration network to discard some neural network units temporarily in accordance with a certain probability. In addition, the use of the simulator can increase the diversity and amount of input features and help eliminate overfitting.

### Method for the evaluation of network performance

A comparative experiment was conducted with Demons [31], a traditional deformable registration method, and VoxelMorph [17], a typical unsupervised deep learning network, to assess the performance of HSCN-Net in the registration of brain CT images.

First, two images were randomly selected from the testing set and defined as the reference image and the moving image. Then, the reference–moving image pair was fed into the network to obtain the deformed image. Finally, qualitative and quantitative analyses were carried out to evaluate the registration accuracy, robustness, and efficiency of HSCN-Net.

Qualitative analysis was performed by visually assessing the registered images. The analysis included assessing the consistency of the anatomical structures and smoothness of the deformations in the registered images.

Quantitative analysis involved metrics, such as EPE, NMI [32], Dice [33] coefficient, and registration time. EPE was used to measure the alignment accuracy between the predicted deformation fields and the gold-standard deformation fields. Given that obtaining the gold standards of the dense deformation fields in image registration was very difficult in practice, this study used anatomical feature point-based sparse deformation vectors to assess EPE differences. First, all of the images in the testing set were manually marked with some typical structural points. The structural points included the fixed points of ventricular horns, the sulcal intersection, etc. Then, the deformation vector for each structural point pair could be computed by transforming the structural point from the moving image space to the reference image space. Finally, EPE could be measured by comparing the gold standard of sparse deformation vectors and the predicted sparse deformation vectors. NMI was used to quantify the correlation between the predicted image and the reference image (ranged from 0 to 1). Large values reflect strong correlation between the predicted image and the reference image and vice versa. The Dice coefficient was utilized to quantify the degree of the overlap between the MCA blood supply area in the predicted and reference images. The gold standards of the MCA blood supply area were annotated by senior neuroradiologists. Moreover, the running time of the three algorithms was statistically analyzed. Paired *t* test was used to compare the registration accuracy and running time of different registration methods, and a *p* value less than 0.05 was considered statistically significant. Refer to Eq. (2) for the definition of EPE.

## Data Availability

The datasets generated and/or analyzed during the current study are not publicly available due to hospital information protection mechanism, but are available from the corresponding author on reasonable request.

## Abbreviations

CT: Computed tomography
ACVD: Acute cerebrovascular disease
EPE: Endpoint error
NMI: Normalized mutual information
MCA: Middle cerebral artery
AIS: Acute ischemic stroke
ASPECTS: Alberta Stroke Program Early CT Score
3D: Three-dimensional
MR: Magnetic resonance
CNNs: Convolutional neural networks
HSCN-Net: Hybrid supervised CNN
